# Evaluation of the effect of a new clinical reasoning curriculum in a pre-clerkship clinical skills course

**DOI:** 10.1007/s40037-020-00566-4

**Published:** 2020-02-13

**Authors:** Arati Kelekar, Nelia Afonso

**Affiliations:** 1 Oakland University William Beaumont School of Medicine, 586 Pioneer Dr, 48309 Rochester, MI USA; 2 Department of Internal Medicine, Beaumont Health System, 3601 West Thirteen Mile Road, 48073 Royal Oak, MI USA

**Keywords:** Clinical reasoning, Clinical skills, Program evaluation, Undergraduate medical education

## Abstract

**Introduction:**

Clinical reasoning is often not explicitly taught to novice medical students. Pre-clerkship clinical skills courses are an ideal venue to teach the clinical reasoning process. The aim of the study was to evaluate the impact of a preclinical clinical reasoning curriculum through an end-of-semester objective structured clinical examination.

**Methods:**

This study was conducted through our longitudinal clinical skills course.

Second year medical (M2) students who received the clinical reasoning curriculum in 2018 formed the study cohort. M2 students from the previous year, who did not have the clinical reasoning curriculum, formed the comparison cohort. Several modalities were used to teach clinical reasoning including whole case approach, serial cue approach, self-explanation of pathophysiological mechanisms and comparison of closely related diagnoses. The students interviewed a standardized patient and documented the history along with three likely diagnoses.

**Results:**

Students in the study cohort achieved higher scores on differential diagnosis (1.98 vs. 1.64 in the comparison cohort, *p* < 0.001). There was no statistically significant difference in the frequency of relevant symptoms queried between the study and comparison cohorts (3.74 vs. 3.34, *p* > 0.05).

**Discussion:**

Our study confirms that the introduction of clinical reasoning in a pre-clerkship clinical skills curriculum increases students’ ability to select relevant symptoms and provides them with a roadmap for expanding their differential diagnoses.

## Background

Clinical reasoning has been defined as the ability to sort through a cluster of features presented by a patient and accurately assign a diagnostic label, with the development of an appropriate treatment strategy as the end goal [1]. Although a core clinical skill, clinical reasoning is difficult to teach. It is complex and learned through experience. Clinician instructors practice it subconsciously, often making it challenging for students to master. The introduction of structured clinical reasoning courses imparting this education in medical school curricula is a relatively recent occurrence [2]. During the preclinical years, medical students are taught communication skills and rudimentary principles of formulating a diagnosis through history taking and clinical examination. During clerkships, students are expected to have adequate foundational knowledge to apply clinical reasoning skills at the bedside. Students who may not have received adequate preclinical clinical reasoning training struggle to grasp these concepts and a significant proportion of these students graduate with suboptimal diagnostic reasoning abilities [3, 4].

Clinical skills courses are an ideal venue to teach the clinical reasoning process. Teaching clinical reasoning at this stage encourages students to integrate basic science knowledge and patient-centered communication skills with clinical reasoning [1, 5]. Clinicians who teach clinical reasoning in clinical skills courses can do so at a different pace compared with a busy clinical setting. These clinicians are best equipped to take students through the process that simulates bedside teaching, making the connections between communication and physical exam skills with clinical reasoning [6].

Sixty-eight percent of US medical schools use a clinical reasoning approach to teach the physical exam [7]. Our clinical skills course did not include a formal clinical reasoning curriculum. It was our observation that students routinely performed unfocused symptom query during the initial patient encounter and faltered in the provision of diagnostic justification.

There is currently no well-defined approach to teaching clinical reasoning to pre-clinical students. There is preliminary evidence that the popularly used serial-cue approach (information is provided serially and only after the questions arising from previously provided information are answered) may not be the ideal modality for teaching clinical reasoning to early level students [8]. It has been suggested that the whole case approach (students are given all the relevant data and expected to identify the findings to support their diagnosis) may be beneficial to translate their nascent pathophysiological knowledge into diagnostic labels [9]. Al Rumayyan et al. have reported that the hypothetico-deduction method might be slightly better than the self-explanation approach [10].

We designed a curriculum that included various strategies (whole case, serial cue, comparison of closely related diagnosis and linking clinical reasoning to pathophysiological knowledge) as well as multiple examples to maximize the availability of concepts and knowledge that have been previously reported to be of benefit [11, 12]. We hypothesized that novice students who experienced the new clinical reasoning curriculum would have a greater ability to select relevant symptoms as a result of early consideration of a differential diagnosis—more in alignment with that of an expert clinician.

## Methods

### Setting and participants

This study was approved by the Oakland University Institutional Review Board, IRB net 1150684‑1. The study was carried out in accordance with the Declaration of Helsinki including, but not limited to, there being no potential harm to participants, that the anonymity of participants was guaranteed, and that informed consent of participants was obtained.

Participants were enrolled in our longitudinal clinical skills course. Consenting second year medical (M2) students (*n* = 101, clinical reasoning curriculum) from academic year 2017–2018 who received the new curricular intervention formed the study cohort, while consenting students from the previous year without the curricular intervention (*n* = 97, no clinical reasoning in the curriculum), formed the comparison cohort.

### Curriculum description

#### Faculty development

Three new sessions (a total of 6 hours) were developed and introduced in the second year clinical skills course. Five clinical faculty associated with the clinical skills course received facilitator guides that included instruction and prompts for the case discussion for all the sessions as well as grading criteria. The facilitator guides highlighted the need for faculty to encourage students to provide justification for their selected diagnoses. The importance of eliciting pertinent symptoms in the diagnostic justification was stressed. All three sessions had assignments which were submitted through the Learning Space® platform for learner performance assessment. Faculty graded each of these assignments and provided students feedback on flawed reasoning.

#### Curricular intervention

##### Session 1: Clinical case presentation—whole case approach

Pairs of students interviewed a standardized patient presenting with one of four non-specific chief complaints (fatigue, weight loss, dizziness, fever). The student pairs formulated a preliminary differential diagnosis and presented these cases to faculty in small groups. Students were asked to recommend hypothesis-driven physical examination maneuvers. Faculty provided physical examination findings, guided the development of the differential diagnosis, and discussed appropriate diagnostic and treatment options.

##### Session 2: Clinical case discussion—serial-cue approach

Two relatively complex cases were presented to the entire class and information related to the case was provided serially. After elaboration on the chief complaint, information on specific symptoms (relevant symptom query) and subsequent case-related details were provided on request. Students worked in small groups and with each request, students were asked to verbally clarify their thought processes before additional information was provided. Faculty addressed misconceptions during these discussions and encouraged students to identify elements in the history that supported or disproved the differential diagnosis. Students then chose the physical exams they would perform. Additional data were provided before finalization and prioritization of the differential diagnoses.

##### Session 3: Online case assignment—self-explanation of pathophysiological mechanisms

Students independently completed an open-book online case assignment which required explanations for the pathophysiological basis of presenting elements in the history, physical examination, and laboratory findings.

During all of these interactive sessions, students were encouraged to compare and contrast closely related diagnoses with evidence to support the final diagnosis and to refute a symptomatically similar diagnosis.

#### Curriculum evaluation

##### Case scenario

The OSCE case involved a middle-aged woman with a previous history of ischemic heart disease, venous thromboembolism and anxiety presenting with episodic palpitations and dizziness. The standardized patient revealed the symptoms of weight loss and diaphoresis or heat intolerance only if specifically asked.

##### Development of clinical reasoning assessment criteria for OSCE post-encounter documentation

Sixteen experienced primary care clinicians with a teaching background were asked to determine relevant symptom query elements and develop a prioritized differential diagnosis for this scenario. Relevant symptom query was defined as the most commonly chosen symptoms (9 symptoms) selected by the majority of physicians and used to score this section of the write-up (Tab. 1). The top 5 diagnoses identified by the majority of the clinicians were considered high priority diagnoses and used to score the differential diagnosis section of the write-up (Tab. 2).

**Table 1:** Relevant symptom query elements (based on clinician responses)

Relevant symptom query^a^	Study cohort (*n* = 101)	Comparison cohort (*n* = 97)	*p*-value
Shortness of breath/dyspnea	65 (64.36%)	88 (82.24%)	0.0035
Chest pain	57 (56.44%)	69 (64.49%)	0.2351
Syncope/presyncope	32 (31.68%)	28 (26.17%)	0.3802
Orthopnea/PND	34 (33.66%)	33 (30.84%)	0.6633
Lower extremity Swelling/edema	41 (40.59%)	45 (42.06%)	0.8305
Diaphoresis/heat intolerance	53 (52.48%)	27 (25.23%)	<0.0001
Weakness or numbness	20 (19.80%)	14 (13.08%)	0.1904
Weight changes	51 (50.50%)	32 (29.91%)	0.0023
Fever	25 (24.75%)	21 (19.63%)	0.3733
*Average number of relevant symptoms queried*	* 3.74*	* 3.34*	*p* *>0.05*

*CR* clinical reasoning, *PND* paroxysmal nocturnal dyspnea

*Study cohort* students with clinical reasoning in the curriculum; *comparison cohort* students not taught clinical reasoning

^a^Organized in the frequency of clinician preference

**Table 2:** High priority differential diagnosis (based on clinician responses)

High priority differential diagnosis^a^	Study cohort (*n* = 100)	Comparison cohort (*n* = 97)	*p*-value
Atrial fibrillation	42 (42.00%)	40 (41.24%)	0.9135
Hyperthyroidism	52 (52.00%)	21 (21.65%)	<0.0001
Acute congestive heart failure	25 (25.00%)	31 (31.96%)	0.2790
Anxiety attack	51 (51.00%)	36 (37.11%)	0.04970
Acute pulmonary embolism	30 (30.00%)	47 (48.45%)	0.0008
*Average score on high priority differential diagnosis* *Maximum: 3 points*	* 1.98 (0.79)*	* 1.64 (0.92)*	*p* *<0.001*

*Study cohort* students with clinical reasoning in the curriculum; *comparison cohort* students not taught clinical reasoning

^a^Organized in the frequency of clinician preference

##### Evaluation of OSCE post-encounter documentation 

The students interviewed a standardized patient and were asked to document a comprehensive history and list their top 3 differential diagnoses. The OSCE case which was administered to the students in the class of 2018 (study cohort with clinical reasoning in the curriculum, current M2) was identical to that administered to the class of 2017 (comparison cohort not taught clinical reasoning, M2 in the previous year). The post-encounter documentation of consenting students was evaluated using the schema as follows. Students were given points for the appropriate symptom query and generation of differential diagnoses. They received 1 point for each relevant symptom recorded in the history of present illness (maximum 9 points). They were asked to list three possible diagnoses and received 1 point for each high priority differential diagnosis (maximum 3 points). The assessment for this project was independent of that used to determine the student’s grade for the OSCE.

##### Statistical analysis

All analysis was performed in SAS 9.4 (SAS Institute Inc., Cary, NC, USA). *P*-values < 0.05 were considered statistically significant. Categorical variables show frequencies with percentages in parentheses. Two samples independent T‑tests are used to compare the average number of relevant symptoms queried and average score on high priority differential diagnoses between the study cohort with clinical reasoning in the curriculum and the comparison cohort not taught clinical reasoning. Bootstrapped standard errors with 500 replicates were used. Relevant symptoms queried and high priority differential diagnosis variables are compared between the study and comparison cohort using Chi-square tests.

## Results

All consenting students completed the history section (*n* = 101 for study cohort with clinical reasoning in curriculum; *n* = 97 for comparison cohort not taught clinical reasoning). At least one diagnosis was entered in the write-up by 100 students in the study cohort and 97 students in the comparison cohort. These were included in the analysis for the high priority differential diagnosis section. The average number of relevant symptoms queried was 3.74 by students in the study cohort versus 3.34 by students in the comparison cohort (*p* > 0.05). The average high priority differential diagnosis score was 1.98 points for students in the study cohort versus 1.64 points for students in the comparison cohort (*p* < 0.001). Both cohorts of students asked about cardiac and respiratory symptoms—chest pain, dyspnea, syncope, orthopnea, paroxysmal nocturnal dyspnea and pedal edema. More students in the study cohort asked about thyroid symptoms including weight changes and heat intolerance or diaphoresis (Tab. 1).

The top 5 high priority differential diagnoses generated by students in the clinical reasoning curriculum mirrored those entertained by the clinicians. However, a smaller number of students in this cohort considered and picked each of the top 3 clinician diagnoses. Students in the study cohort had a broader differential diagnosis as compared with students in the comparison cohort who confined their possibilities to cardiac and pulmonary causes (Tab. 2). Although not considered a high priority diagnosis, a significant number of students in the comparison cohort listed acute MI as a possibility, compared with the study cohort (43% vs 25%, *p* = 0.006).

## Discussion

This paper describes the impact of a curriculum for teaching clinical reasoning to preclinical medical students using several different strategies. Our study confirms that the introduction of a clinical reasoning curriculum in a pre-clerkship clinical skills course provides pre-clerkship students with a roadmap for expanding their differential diagnoses.

As suggested by Kassirer (2010) and Rencic (2011), utilization of clinical cases with multiple clinical reasoning aspects enhanced the opportunity to emphasize various concepts in clinical reasoning: serial cue approach for early hypothesis generation in straightforward cases; online cases for scaffolding to preexisting pathophysiological knowledge; and complex whole cases with multi-organ system differential diagnosis consideration [12, 13]. This format exposed students to a repertoire of cases that strengthened the acquisition and development of ‘illness scripts’ and non-analytical clinical reasoning [14].

A unique component of this curriculum was the interaction with clinical faculty for coaching and feedback as well as for assessment. In addition to explaining their own rationale whilst analyzing the cases, faculty were available to guide students as they developed the differential diagnosis and played an indispensable role in recognizing their erroneous reasoning. Although a non-work-based assessment, the OSCE using standardized patients tested higher order thinking skills—not only recall, but the application of knowledge as well. This format, similar to a real patient encounter, encouraged students to gather the appropriate data and arrive at a diagnosis as recommended by Daniel et al. [15].

Students in the study cohort, with clinical reasoning in the curriculum, were more likely to identify symptoms not anatomically related to the organ system of the chief complaint (i.e. weight loss, heat intolerance, diaphoresis) compared with students in the comparison cohort, who were more likely to identify anatomically related symptoms (shortness of breath and chest pain). Similarly, the differential diagnosis by the clinical reasoning students was broader and included hyperthyroidism and anxiety (anatomically unrelated to the chest complaint) while diagnoses such as pulmonary embolism and myocardial infarction were more often considered by the comparison cohort. This suggests that the comparison cohort may have failed to consider alternative diagnoses after the initial impression was formed.

The diagnoses provided by the clinical reasoning students mirrored those entertained by the clinicians.

This is a single institutional study and the authors do not have quantitative data on which of the methods is better for teaching clinical reasoning or whether the increased faculty time spent with coaching and feedback improved clinical reasoning skills. In addition, this was a single station OSCE, hence this limits our ability to extrapolate student performance in selecting relevant symptoms with diverse patient scenarios.

In light of the demonstrated benefits of the above approach, we plan to expand this curriculum during the M1 year. Our future goal is to evaluate for any long-term effects with respect to the demonstration of clinical reasoning during patient care in the clerkship years.
